# A novel prognostic index for oral squamous cell carcinoma patients with surgically treated

**DOI:** 10.18632/oncotarget.14821

**Published:** 2017-01-26

**Authors:** Fa Chen, Yujie Cao, Jiangfeng Huang, Lingjun Yan, Lisong Lin, Fengqiong Liu, Fangping Liu, Junfeng Wu, Yu Qiu, Lin Cai, Baochang He

**Affiliations:** ^1^ Department of Epidemiology and Health Statistics, School of Public Health, Fujian Medical University, Fujian, China; ^2^ Key Laboratory of Ministry of Education for Gastrointestinal Cancer, Fujian Medical University, Fujian, China; ^3^ Department of Stomatology, The First Affiliated Hospital of Fujian Medical University, Fujian, China; ^4^ Department of Oral and Maxillofacial Surgery, The First Affiliated Hospital of Fujian Medical University, Fujian, China

**Keywords:** oral squamous cell carcinoma, prognostic prediction model, prognostic index, overall survival, prospective study

## Abstract

This study aims to develop an applicable prognostic index with conventional factors for predicting outcome of patients with oral squamous cell carcinoma (OSCC). We performed a prospective study in a large cohort of 892 OSCC patients in Fujian, China. All patients were randomly divided into a discovery group and validation group. A prognostic index was developed based on β value of each significant variable obtained from the multivariate Cox regression model. The results from discovery and validation set demonstrated thatthe model-4(included clinical stage, tumor differentiation, ill-fitting denture, oral hygiene and cigarette smoking) was the optimal model. The optimal cutoff points of prognostic index (1.88 and 2.80) were determined by X-tile program which categorized all subjects into low, middle and high risk subsets. Patients in high risk group were at the greatest risk of death compared with those in low risk group (HR: 6.02; 95%CI: 4.33-8.38). Moreover, there was a significant tendency of the worse overall survival with the higher prognostic index (*P*trend <0.001). The discriminatory capacity of prognostic index was 0.661(95%CI: 0.621-0.701). This study developed and validated a prognostic index that is an economical and useful tool for predicting the clinical outcomes of OSCC patients in Southeast China. Future randomized trials with larger cohort are required to confirm our results.

## INTRODUCTION

Oral squamous cell carcinomas (OSCC) is the predominant histological type in oral carcinomas and has a rising morbidity and mortality in many countries [1, 2]. Despite many advances in the diagnosis and treatment, long-term survival of OSCC only improved slightly over the past few decades, with 5-year survival rate still around 50% [3, 4]. It is therefore essential to explore prognostic factors which would be better to predict OSCC patients’ prognosis.

Various parameters have been reported in previous studies for predicting the prognosis of OSCC, including clinicopathologic features (clinical stage, tumor differentiation, tumor size, and treatment types, etc.) [5–7], serum biomarkers (C-reactive protein (CRP), neutrophil to lymphocyte ratio (NLR), etc.) [8, 9], human papillomavirus (HPV) infection [10] and patients’ lifestyle factors (smoking, alcohol drinking, oral hygiene, etc.) [11, 12]. However, few studies incorporate these prognostic parameters into a comprehensive index to evaluate the joint contribution to the prognosis of this disease.

Recently, prognostic prediction model with different independent prognostic factors has been proposed and became a promising method for reliable prediction of cancer prognosis. Tertipis et al. [13] utilised HLA class I, CD8+ TILs and clinical characteristics to predict the outcomes of tongue cancer patients. Rietbergen et al. [14] established a prognostic model included HPV infection, comorbidity and nodal stage for oropharyngeal squamous cell carcinoma. Almangush et al. [15] combined tumor budding and depth of invasion into a predictive model for tongue cancer. However, most of previous models contain costly biomarkers and not sufficiently validated with small sample size and short-term follow up.

Therefore, we performed a prospective study with large samples size and long-term follow-up to develop and validate a practical prognostic index (PI) using simple and easily available clinical parameters to predict the outcomes of OSCC patients in Southeast of China.

## RESULTS

The demographic characteristics of discovery group and validation group are listed in Table 1. The 5-year overall survival (OS) rate for all OSCC patients was 67.6% (69.6% for discovery group and 66.0% for validation group). There was no significant difference in demographic characteristics between two groups (all *P* > 0.05).

**Table 1:** Distribution of demographic characteristics between discovery and validation groups

Variables	Discovery group(*n*= 446)	Validation group(*n*= 446)	*P*^b^
Sex			0.158
Female	163(36.55)	143(32.06)	
Male	283(63.45)	303(67.94)	
Age (years)			0.177
<60	242(54.26)	262(58.74)	
≥60	204(45.74)	184(41.26)	
BMI ^a^ (kg/m^2^)			0.385
18.5-23.9	280(62.78)	264(59.19)	
<18.5	71(15.92)	86(19.28)	
≥24	95(21.30)	96(21.53)	
Occupation			0.625
Farmer	174(39.02)	184(41.26)	
Worker	96(21.52)	100(22.42)	
Staff and others	176(39.46)	162(36.32)	
Residence			0.310
Urban	249(55.83)	264(59.19)	
Rural	197(44.17)	182(40.81)	
Education level			0.579
Primary and below	57(12.78)	47(10.54)	
Middle school	325(72.87)	334(74.89)	
College and above	64(14.35)	65(14.57)	
Family history of cancer			0.784
No	376(84.30)	373(83.63)	
Yes	70(15.70)	73(16.37)	

^a^ Body mass index (weight in kg/[height in m]^2^), BMI groups according to the classification criteria for Chinese adults; ^b^ Chi-square test

Table 2 presents the HRs of potential prognosis factors for OSCC in discovery group. Advanced tumour stage was significantly associated with worse survival: the HRs were 4.57 (95% CI: 2.30-9.11) for stage IV, 4.36 (95% CI: 2.15-8.8) for stage III and 2.05 (95% CI: 1.01-4.15) stage II. Moreover, patients with moderate and poor histological differentiation had an increased risk for death (HR: 1.60 (95% CI 1.07-2.39) and 3.19 (95% CI: 1.98-5.15), respectively). Additionally, cigarette smoking, ill-fitting denture and poor oral hygiene also significantly elevated risk of death.

**Table 2:** Univariate analysis of potential prognosis factors for OSCC patients in discovery group

Variables	Number of Censored (%)	Number of death (%)	5-year OS (%)	HR(95 % CI)	Log-rank *P*
Sex					
Female	122(38.01)	41(32.80)	67.91	1.00	0.344
Male	199(61.99)	84(67.20)	71.58	1.20(0.82-1.74)	
Age (years)					
<60	177(55.14)	65(52.00)	72.80	1.00	0.295
≥60	144(44.86)	60(48.00)	66.88	1.21(0.85-1.71)	
BMI (kg/m^2^)					
18.5-23.9	196(61.06)	84(67.20)	69.25	1.00	0.077
<18.5	46(14.33)	25(20.00)	65.78	1.19(0.76-1.86)	
≥24	79(24.61)	16(12.80)	75.03	0.59(0.35-1.01)	
Occupation					
Farmer	113(35.21)	61(48.80)	67.88	1.00	0.399
Worker	62(19.31)	34(27.20)	69.93	0.94(0.62-1.43)	
Staff and others	146(45.48)	30(24.00)	75.87	0.74(0.48-1.15)	
Residence					
Urban	154(47.98)	63(50.40)	71.74	1.00	0.967
Rural	167(52.02)	62(49.60)	68.49	0.99(0.70-1.41)	
Education level					
Primary and below	44(13.71)	13(10.40)	65.56	1.00	0.983
Middle school	229(71.34)	96(76.80)	70.82	1.01(0.57-1.81)	
College and above	48(14.95)	16(12.80)	70.07	1.06(0.51-2.21)	
Family history of cancer					
No	272(84.74)	104(83.20)	72.11	1.00	0.774
Yes	49(15.26)	21(16.80)	68.21	1.03(0.60-1.79)	
Stage					
I	65(20.25)	10(8.00)	88.89	1.00	<0.001
II	109(33.95)	33(26.40)	77.58	2.05(1.01-4.15)	
III	97(30.22)	38(30.40)	58.98	4.36(2.15-8.83)	
IV	50(15.58)	44(35.20)	58.84	4.57(2.30-9.11)	
T stage					
T1	75(23.36)	19(15.20)	89.77	1.00	0.002
T2	126(39.25)	49(39.20)	70.10	1.81(1.07-3.08)	
T3	90(28.04)	33(26.40)	67.48	2.39(1.35-4.23)	
T4	30(9.35)	24(19.20)	50.83	3.00(1.64-5.46)	
N stage					
N0	241(75.08)	70(56.00)	78.57	1.00	<0.001
N1	46(14.33)	26(20.80)	55.48	2.68(1.69-4.25)	
N2-3	34(10.59)	29(23.20)	44.07	3.13(2.02-4.85)	
M stage					
M0	318(99.07)	120(96.00)	70.51	1.00	0.114
M1	3(0.93)	5(4.00)	50.00	2.03(0.83-4.99)	
Histologic grade					
Well	159(49.53)	42(33.60)	81.14	1.00	<0.001
Moderate	132(41.12)	55(44.00)	65.43	1.60(1.07-2.39)	
Poor	30(9.35)	28(22.40)	48.63	3.19(1.98-5.15)	
Treatment					
Surgical	129(40.19)	35(28.00)	67.91	1.00	0.390
Surgical+CT	74(23.05)	28(22.40)	76.80	0.74(0.45-1.23)	
Surgical+RT	37(11.53)	15(12.00)	70.19	0.99(0.54-1.81)	
Surgical+CRT	81(25.23)	47(37.60)	65.90	1.12(0.72-1.74)	
Smoking status					
No	221(68.85)	75(60.00)	70.78	1.00	0.014
Yes	100(31.15)	50(40.00)	68.92	1.57(1.09-2.24)	
Drinking status					
No	254(79.13)	97(77.60)	71.27	1.00	0.574
Yes	67(20.87)	28(22.40)	69.04	1.13(0.74-1.72)	
Oral hygiene					
Well	81(25.23)	24(19.20)	88.34	1.00	0.004
Poor	240(74.77)	101(80.80)	64.59	1.92(1.22-3.02)	
Ill-fitting denture					
No	189(58.88)	78(62.40)	74.22	1.00	0.006
Yes	132(41.12)	47(37.60)	65.26	1.67(1.15-2.42)	
Comorbidity ^a^					
No	161(50.16)	57(45.60)	75.54	1.00	0.124
Yes	160(49.84)	68(54.40)	64.97	1.32(0.93-1.88)	

Abbreviations: CT, chemotherapy; RT, radiotherapy; CRT, chemoradiotherapy.

^a^ Comorbidity, the simultaneous existence of other medical conditions.

Next, we performed a multivariate Cox regression analysis to develop different models by incorporating potentially significant or important factors obtained from Table 2. As shown in Table 3, the model-4 (including tumor stage, histologic grade, cigarette smoking, ill-fitting denture, oral hygiene) showed the highest Harrell's c-statistic (0.743), which had significant differences with model-1 (*P* < 0.001), model-2 (*P* = 0.036) and model-3 (*P* = 0.026), respectively. Moreover, the model-4 had the highest discriminatory ability for 5-year OS, with the lowest Akaike information criterion (AIC) value (1250.115). The above-mentioned data indicated that model-4 was a superior prognostic model for OSCC compared to other models.

**Table 3:** Multivariate analysis of potential prognosis factors in four models in discovery group

Variables	Model-1^a^		Model-2 ^b^		Model-3 ^c^		Model-4 ^d^	
	β	HR(95 % CI)	β	HR(95 % CI)	β	HR(95 % CI)	β	HR(95 % CI)
Stage								
I		1.00		1.00		1.00		1.00
II	0.58	1.79(0.87-3.65)	0.82	2.26(1.10-4.65)	0.61	1.84(0.90-3.76)	0.73	2.08(1.01-4.29)
III	1.35	3.86(1.89-7.90)	1.32	3.73(1.82-7.64)	1.47	4.35(2.11-8.95)	1.25	3.50(1.67-7.31)
IV	1.44	4.24(2.11-8.51)	1.56	4.75(2.36-9.56)	1.48	4.38(2.15-8.91)	1.52	4.58(2.26-9.31)
Histologic grade								
Well		1.00		1.00		1.00		1.00
Moderate	0.43	1.53(1.01-2.32)	0.45	1.57(1.04-2.36)	0.43	1.53(1.01-2.31)	0.46	1.59(1.05-2.40)
Poor	1.20	3.33(2.04-5.43)	1.22	3.39(2.07-5.55)	1.05	2.86(1.74-4.71)	1.28	3.61(2.17-6.03)
Smoking status								
No		1.00						1.00
Yes	0.53	1.69(1.16-2.46)					0.58	1.78(1.13-2.79)
Ill-fitting denture								
No				1.00				1.00
Yes			0.59	1.80(1.18-2.74)			0.56	1.75(1.12-2.74)
Oral hygiene								
Well						1.00		1.00
Poor					0.57	1.76(1.09-2.84)	0.59	1.81(1.12-2.92)
Harrell's c-statistic (95 % CI)		0.709(0.659-0.758)		0.714(0.666-0.762)		0.713(0.666-0.759)		0.743(0.697-0.788)
AIC		1264.644		1264.201		1265.276		1250.115

^a^ Model-1 include age, sex, BMI, comorbidity, treatment, tumor stage, histologic grade and cigarette smoking; ^b^ Model-2 include age, sex, BMI, comorbidity, treatment, tumor stage, histologic grade and ill-fitting denture; ^c^ Model-3 include age, sex, BMI, comorbidity, treatment, tumor stage, histologic grade and oral hygiene; ^d^ Model-4 include age, sex, BMI, comorbidity, treatment, tumor stage, histologic grade, cigarette smoking, ill-fitting denture and oral hygiene.

**Figure 1:**
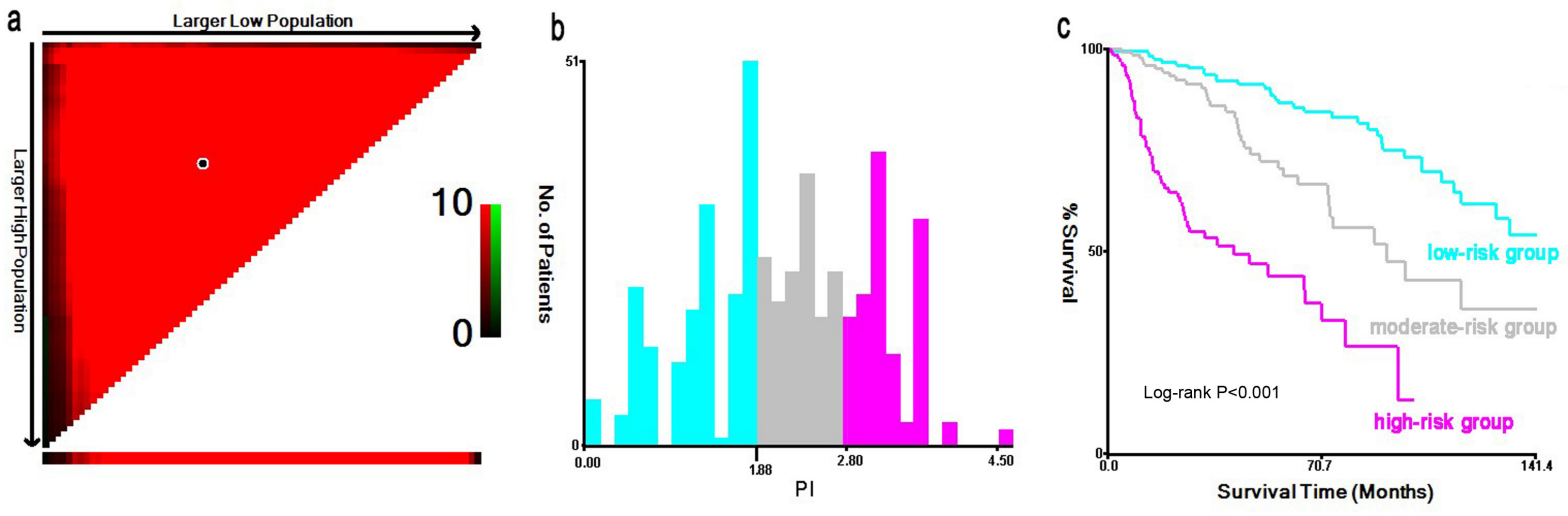
X-tile analysis on the optimal cutoff points of prognostic index for the discovery group The optimal cut-point marked by the black point in the (**a**) is displayed on a histogram of the cohort (**b**), and a Kaplan-Meier plot (**c**).

To further evaluate the prognostic value of model-4, we developed a composite PI according to five significant factors in model-4 (PI = 0.73×stage II +1.25×stage III +1.52×stage IV +0.46×moderate differentiation +1.28×poor differentiation +0.58×cigarette smoking +0.56×ill-fitting denture+0.59×oral hygiene). The median of PI was 2.30 ranging from 0 to 4.53, and the higher the PI, the poorer the survival. Then, the X-tile program determined 1.88 and 2.80 as the optimal cutoff values with the minimum P value (χ^2^ = 85.14, *P* < 0.001), which divided the cohort into low, middle and high risk subsets (Figure 1a-1b). Moreover, patients in high-risk group had the significantly worst OS than low-risk group (*P* < 0.001, Figure 1c; Table 4).

**Table 4:** Association between PI value and the prognosis of patients with OSCC

Variable	Discovery group		Validation group		All patients	
	*N*	HR(95%CI)^a^	*N*	HR(95%CI)^a^	*N*	HR(95%CI)^a^
PI						
Median(quartile)	446	2.30(1.61,2.92)	446	2.33(1.63,3.10)	892	2.30(1.62,3.05)
subgroup						
0-1.88 (low risk)	184	1.00	180	1.00	362	1.00
1.89-2.80 (moderate risk)	136	1.90(1.14,3.16)	128	2.48(1.52,4.04)	266	2.06(1.46,2.88)
≥2.81 (high risk)	126	7.47(4.57,12.22)	138	5.30(3.28,8.56)	264	6.21(4.43,8.70)
P for trend		<0.001		0.005		<0.001
AUROC		0.639(0.581,0.697)		0.677(0.623,0.731)		0.661(0.621,0.701)

**Figure 2:**
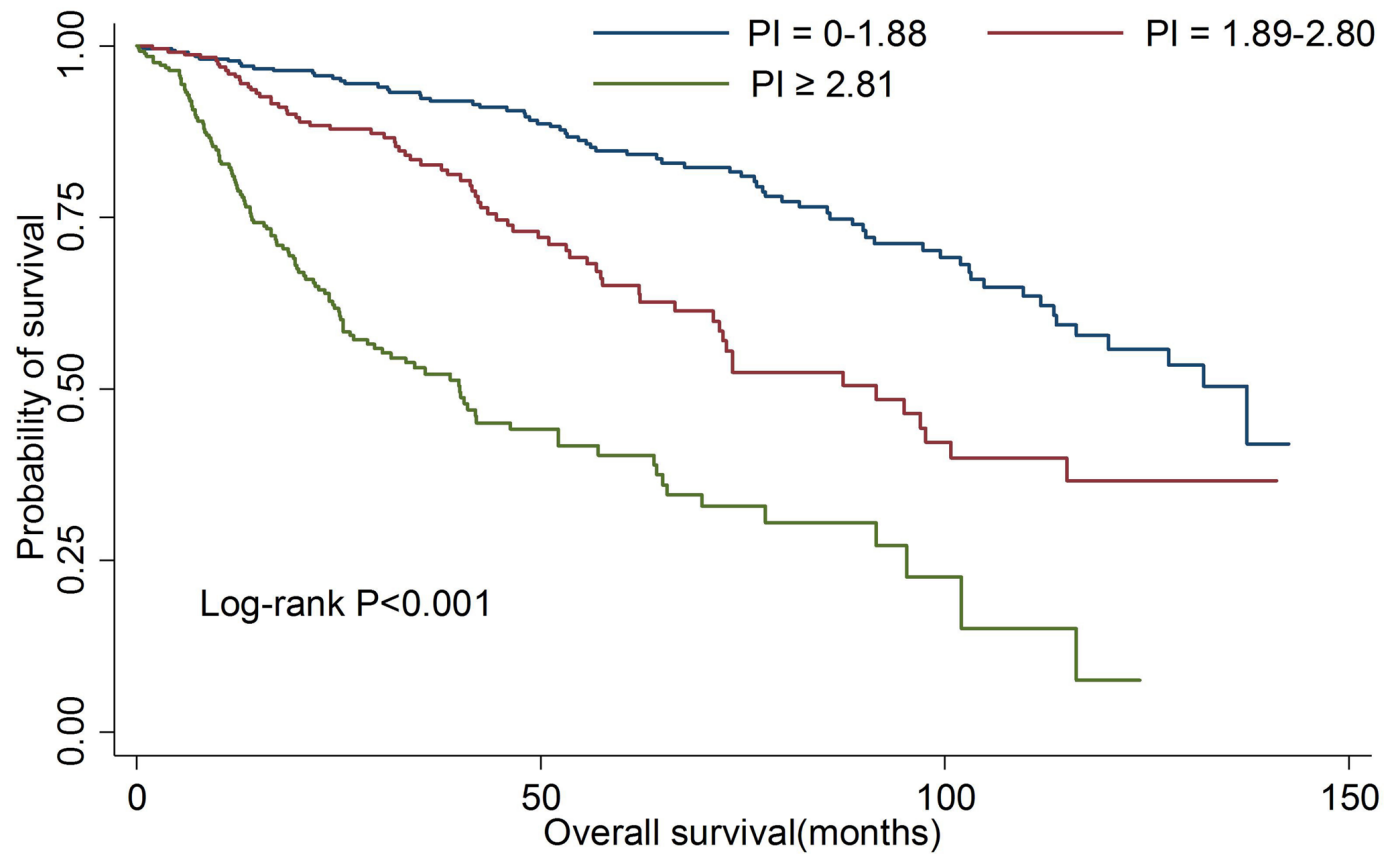
Kaplan-Meier curves for overall survival according to prognostic index in validation group

Furthermore, we re-examined the four model to validate the above results in an independent set. The model-4 still had the highest Harrell's c-statistic (0.738, 95%CI: 0.695-0.781; data not shown). Moreover, no significant difference of Harrell's c-statistic was observed between discovery group and validation group (*P* > 0.05). The overall survival rates were significantly different in the different PI groups, with *P* < 0.001 by log-rank test (Figure 2).

**Figure 3:**
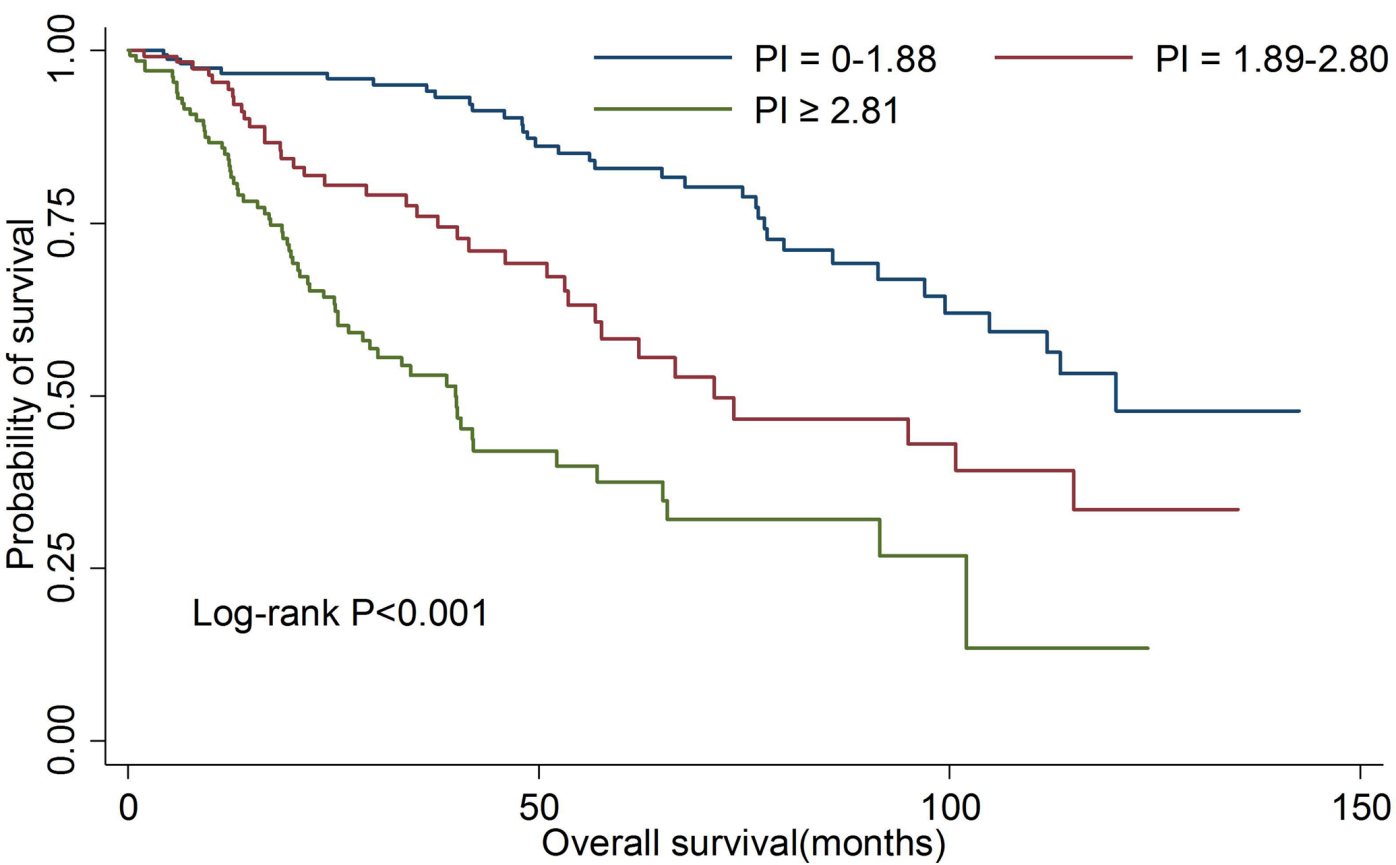
Kaplan-Meier curves for overall survival according to prognostic index in all patients

We then estimated the clinical prediction value of PI in all OSCC patients. As shown in Table 4, patients in high risk group were at the greatest risk of death compared with those in low risk group (HR: 6.02; 95%CI: 4.33-8.38). There was also a significant linear trend in the risk (*P*trend < 0.001). The 5-year OS rates for three risk subsets were 84.6%, 65.2% and 40.7%, respectively. Kaplan-Meier curve analysis also showed the similar results (Figure 3). For death prediction, the area under the receiver operating characteristic curve (AUROC) of PI was 0.661(95%CI: 0.621-0.701).

## DISCUSSION

In this large cohort prospective study, we developed and validated a novel prognostic index based on a superior prognostic model for OSCC. The model consisted of five significant factors: tumour stage, histologic grade, cigarette smoking, ill-fitting denture and oral hygiene. Moreover, there was a significant tendency of the worse OS with the higher PI value.

Among the significant prognostic factors in the present study, high clinical stage and poor tumor differentiation showed strong associations with risks of mortality among OSCC patients, which are traditional prognostic predictors and have been well-documented in many previous studies [16]. Cigarette smoking was found to be an independent predictor of survival in OSCC, which is consistent with other studies [11]. The carcinogens contained in tobacco smoke may affect the prognosis of OSCC by changing the oral microenvironment and inducing recurrent inflammatory responses [17, 18]. Additionally, ill-fitting dentures could give rise to chronic mucosal irritation, and subsequently cause trauma and recurrent oral inflammation, which results in release of inflammatory mediators and growth factors that may be associated with a high rate of recurrence and a poor response to radiotherapy [19, 20]. The possible mechanism of poor oral hygiene on the worse outcome of OSCC may be that poor oral hygiene may induce the imbalance of oral flora and lead to postoperative inflammation easily, which may activate the specific chemokine that can regulate cell proliferation, survival and metastasis [20, 21].

Although several previous studies have created prognostic prediction models with different discriminatory ability for the prognosis of oral cancer [13–15, 22], the limitations of small sample size and short-term follow-up could not be avoided. Moreover, some of the parameters incorporated are not routine clinical measurements, which may limit the use of these models in clinical practice. In this study, we incorporated five conventional prognostic parameters to develop an easy applied prognostic prediction model, which is a credible tool to evaluate outcome as these variables are inexpensive and can be simple to calculate by physicians. The PI value with high discriminatory ability would better assess clinical prognosis and facilitate the development of individual treatment.

Nevertheless, there are several limitations in our study. First, we only included clinical features and lifestyle factors in the model, and did not consider serum markers and other novel biomarkers, which may limit the discriminatory capacity of PI within a certain range. However, taking more factors into account would increase the additional measurement cost, and make the model cannot gain widespread acceptance. Hopefully, other routine clinical measurements will also be considered to improve our model in future studies. Second, the variables were measured only at the start of the study but not at follow-up. It therefore needs further studies to get a further validation.

In conclusion, we developed and validated a prognostic index determined by a superior prognostic model for predicting the clinical outcomes of OSCC patients in Southeast China. The PI may be an economical, widely available and useful tool to plan therapeutic strategies and guide the schedule of individualized treatment. Future randomized trials with larger cohort are required to confirm our results.

## MATERIALS AND METHODS

### Study design and population

All study subjects were consecutively recruited between December 2003 and December 2015 at The First Affiliated Hospital of Fujian Medical University (Fujian, China). As described previously [23], subjects were included if they met the following conditions: (1) all patients were primary OSCC patients with histologically diagnosed; (2) all patients had underwent surgical resection; (3) all patients are all Chinese Han population who aged 20-80 years and reside in Fujian Province. Those who had other synchronous malignancies, or were diagnosed with recurrent oral cancer or metastasized cancer, were excluded from this study. The final analysis dataset consisted of 892 OSCC patients (26 lip, 447 tongue, 122 gingiva, 63 palate, 119 buccal, 66 floor of mouth, and 49 unspecified or overlapping). Informed consents were obtained from all patients. Our study was approved by the Institutional Review Board (IRB) of Fujian Medical University (Fuzhou, China) and conducted in line with the ethical standards described in the Declaration of Helsinki.

### Data collection

Information on demographics and lifestyle habits were obtained by trained interviewers through face-to-face interview with a structured questionnaire. Clinical characteristics (clinical stage, treatment types, tumor differentiation, histological types, comorbidity, etc.) were collected from medical records. Smokers were defined as those who had smoked at least 100 cigarettes during their lifetime. Oral hygiene status at diagnosis was evaluated by physicians through oral inspection. Ill-fitting denture refers to the denture with sharp or rough surfaces, or having overextended flanges, or lacking of stability and retention.

### Prospective follow-up

Follow-up data were obtained by telephone interview and medical records of readmission. Telephone interview was conducted every six months until the patient died or the final follow-up dated June 31, 2016. 892 OSCC patients were followed up for 3,124 person years. The median follow-up time was 70.7 months for all patients. During the follow-up period, a total of 271(30.38%) patients died (262 deaths from OSCC), 113(12.67%) were lost to follow up and 508(56.95%) were still alive. Those who were still alive or who died from other causes or who were lost to follow up were considered to be censored data. The primary endpoint was OS, which was calculated from the date of diagnosis to the date of death from any cause or the last follow up.

### Statistical analysis

Two-stage analyses were performed through randomly divided 892 patients into a discovery set (*n* = 446) and a validation set (*n* = 446). Univariate Cox regression model was utilized to estimate the associations between variables and OS. Four different multivariate Cox regression models were used to assess independent factors in OSCC prognosis. The superior prognostic model was evaluated and validated using Harrell's c-statistic and AIC. Then, a PI for OSCC was developed based on β value of each significant variable obtained from the superior model. The optimal cutoff points PI were identified using the X-tile program [24]. Trend test for PI was performed by entering PI subgroup as continuous variable in the regression model, and P value was obtained from Wald chi-square test. AUROC was calculated to evaluate the clinical prediction value and discriminatory capacity of PI. Survival curves were generated by the Kaplan-Meier method, and compared by the log-rank test. Statistical significance was defined when *P* < 0.05. All statistical analyses were conducted with R software (version 3.1.1).

## References

[1] Zhang SK, Zheng R, Chen Q, Zhang S, Sun X, Chen W (2015). Oral cancer incidence and mortality in China, 2011. Chin J Cancer Res.

[2] van Dijk BA, Brands MT, Geurts SM, Merkx MA, Roodenburg JL (2016). Trends in oral cavity cancer incidence, mortality, survival and treatment in the Netherlands. Int J Cancer.

[3] Freier K, Engel M, Lindel K, Flechtenmacher C, Muhling J, Hassfeld S, Hofele C (2008). Neoadjuvant concurrent radiochemotherapy followed by surgery in advanced oral squamous cell carcinoma (OSCC): a retrospective analysis of 207 patients. Oral Oncol.

[4] Dillon JK, Brown CB, McDonald TM, Ludwig DC, Clark PJ, Leroux BG, Futran ND (2015). How does the close surgical margin impact recurrence and survival when treating oral squamous cell carcinoma?. J Oral Maxillofac Surg.

[5] Rodrigues PC, Miguel MC, Bagordakis E, Fonseca FP, de Aquino SN, Santos-Silva AR, Lopes MA, Graner E, Salo T, Kowalski LP, Coletta RD (2014). Clinicopathological prognostic factors of oral tongue squamous cell carcinoma: a retrospective study of 202 cases. Int J Oral Maxillofac Surg.

[6] Liu F, Chen F, Huang J, Yan L, Liu F, Wu J, Qiu Y, Zheng X, Zhang R, Lin L, He B (2017). Prospective study on factors affecting the prognosis of oral cancer in a Chinese population. Oncotarget.

[7] Zhong LP, Zhang CP, Ren GX, Guo W, William WN, Sun J, Zhu HG, Tu WY, Li J, Cai YL, Wang LZ, Fan XD, Wang ZH (2013). Randomized phase III trial of induction chemotherapy with docetaxel, cisplatin, and fluorouracil followed by surgery versus up-front surgery in locally advanced resectable oral squamous cell carcinoma. J Clin Oncol.

[8] Fang HY, Huang XY, Chien HT, Chang JT, Liao CT, Huang JJ, Wei FC, Wang HM, Chen IH, Kang CJ, Huang SF (2013). Refining the role of preoperative C-reactive protein by neutrophil/lymphocyte ratio in oral cavity squamous cell carcinoma. Laryngoscope.

[9] Chen F, Lin L, Yan L, Qiu Y, Cai L, He B (2017). Preoperative neutrophil-to-lymphocyte ratio predicts the prognosis of oral squamous cell carcinoma: a large-sample prospective study. J Oral Maxillofac Surg.

[10] Duray A, Descamps G, Decaestecker C, Remmelink M, Sirtaine N, Lechien J, Ernoux-Neufcoeur P, Bletard N, Somja J, Depuydt CE, Delvenne P, Saussez S (2012). Human papillomavirus DNA strongly correlates with a poorer prognosis in oral cavity carcinoma. Laryngoscope.

[11] Kawakita D, Hosono S, Ito H, Oze I, Watanabe M, Hanai N, Hasegawa Y, Tajima K, Murakami S, Tanaka H, Matsuo K (2012). Impact of smoking status on clinical outcome in oral cavity cancer patients. Oral Oncol.

[12] Morais MO, Elias MR, Leles CR, JC Dourado Pinezi, Mendonca EF (2016). The effect of preventive oral care on treatment outcomes of a cohort of oral cancer patients. Support Care Cancer.

[13] Tertipis N, Hammar U, Nasman A, Vlastos A, Nordfors C, Grun N, Ahrlund-Richter A, Sivars L, Haeggblom L, Marklund L, Hammarstedt-Nordenvall L, Chaturvedi AK, Munck-Wikland E (2015). A model for predicting clinical outcome in patients with human papillomavirus-positive tonsillar and base of tongue cancer. Eur J Cancer.

[14] Rietbergen MM, Witte BI, Velazquez ER, Snijders PJ, Bloemena E, Speel EJ, Brakenhoff RH, Kremer B, Lambin P, Leemans CR (2015). Different prognostic models for different patient populations: validation of a new prognostic model for patients with oropharyngeal cancer in Western Europe. Br J Cancer.

[15] Almangush A, Coletta RD, Bello IO, Bitu C, Keski-Santti H, Makinen LK, Kauppila JH, Pukkila M, Hagstrom J, Laranne J, Tommola S, Soini Y, Kosma VM (2015). A simple novel prognostic model for early stage oral tongue cancer. Int J Oral Maxillofac Surg.

[16] Kreppel M, Nazarli P, Grandoch A, Safi AF, Zirk M, Nickenig HJ, Scheer M, Rothamel D, Hellmich M, Zoller JE (2016). Clinical and histopathological staging in oral squamous cell carcinoma - Comparison of the prognostic significance. Oral Oncol.

[17] Kumar PS, Matthews CR, Joshi V, de Jager M, Aspiras M (2011). Tobacco smoking affects bacterial acquisition and colonization in oral biofilms. Infect Immun.

[18] Schlage WK, Iskandar AR, Kostadinova R, Xiang Y, Sewer A, Majeed S, Kuehn D, Frentzel S, Talikka M, Geertz M, Mathis C, Ivanov N, Hoeng J (2014). *In vitro* systems toxicology approach to investigate the effects of repeated cigarette smoke exposure on human buccal and gingival organotypic epithelial tissue cultures. Toxicol Mech Methods.

[19] Chen F, He BC, Yan LJ, Qiu Y, Lin LS, Cai L (2017). Influence of oral hygiene and its interaction with standard of education on the risk of oral cancer in women who neither smoked nor drank alcohol: a hospital-based, case-control study. Br J Oral Maxillofac Surg.

[20] Feller L, Altini M, Lemmer J (2013). Inflammation in the context of oral cancer. Oral Oncol.

[21] Perera M, Al-Hebshi NN, Speicher DJ, Perera I, Johnson NW (2016). Emerging role of bacteria in oral carcinogenesis: a review with special reference to perio-pathogenic bacteria. J Oral Microbiol.

[22] McBride SM, Busse PM, Clark JR, Wirth LJ, Ancukiewicz M, Chan AW (2014). Long-term survival after distant metastasis in patients with oropharyngeal cancer. Oral Oncol.

[23] Chen F, He B, Yan L, Qiu Y, Lin L, Cai L (2017). FADS1 rs174549 Polymorphism May Predict a Favorable Response to Chemoradiotherapy in Oral Cancer Patients. J Oral Maxillofac Surg.

[24] Camp RL, Dolled-Filhart M, Rimm DL (2004). X-tile: a new bio-informatics tool for biomarker assessment and outcome-based cut-point optimization. Clin Cancer Res.

